# Perception of Paralytic Ileus on Viewpoint of Avicenna

**Published:** 2017-01

**Authors:** Ebrahim KHADEM, Mahboobeh SHIRAZI, Roja RAHIMI, Sodabeh BIOOS, Fereshteh GHORAT

**Affiliations:** 1. Dept. of Traditional Medicine, School of Traditional Medicine, Tehran University of Medical Sciences, Tehran, Iran; 2. Dept. of Gynecology, School of Traditional Medicine, Tehran University of Medical Sciences, Tehran, Iran

## Dear Editor-in-Chief

Paralytic ileus is type of functional obstruction caused by the lack of intestinal peristalsis without any mechanical obstruction. This interference of the flow of intestinal contents often leads to intestinal obstruction. Paralytic ileus may be classified into postoperative, inflammatory, metabolic, neurogenic, and drug-induced (1). The incidence of paralytic ileus is high especially after abdominal surgery. Postoperative ileus is one of the most important factors affecting early recovery and is a cause of morbidity surgery and prolonged hospital stay (2). Despite the huge cost to the health system, there have been very few advances in clinicians approach to ileus. In fact, the main etiology of ileus is unknown and requires a multifactorial therapeutic approach (3). An alternative approach could help certain themes ignored by new studies.

Iranian traditional medicine is one of the oldest traditional medicines. Avicenna (980–1037AD) was a great Iranian clinician and philosopher. He in his famous book, Qanun of Medicine thoroughly has reviewed all of the medical science of ancient Greek and Muslim scholars (4, 5).

Based on Iranian traditional medicine, paralytic ileus, type of functional intestinal obstruction, has been described under title of Gholonj disease. This chapter deals with the physiopathology and management of the gastrointestinal obstructive disorders in Iranian traditional medicine (6, 7). Gholonj is a general terms applied to any abdominal pain along with no gas passing and defecation. Avicenna considered the simple abdominal pain different from Gholonj; and that condition has investigated under the title of “Maghs”. The different aspect of these two diseases is passing or non-passing of intestinal contents. On the other hand, Gholonj includes a range of intestinal diseases that the most important characteristic is insufficiency of intestinal movement and inability to pass flatus and defection. The main place of involving in Gholonj is colon and the name of Gholonj has derived from Gholon that is the Arabic term of colon. The symptoms of Gholonj are a significant abdominal pain, nausea, and vomiting, abdominal distention, inability to defecation or gas passing and decreased appetite. In intensive cases of disease may are seen inability to urinate, thirst, chills and disturbance of consciousness. Avicenna believes the most important physiopathology of this disease is intestinal obstruction.

Golonj is classified based on the etiology of obstruction (mechanical or non-mechanical) to six categories (Table 1). Non-mechanical obstruction can be created because of accumulation of gas or secretion of some material into intestinal lumen. In addition, Avicenna believes that Gholonj can be developed secondary to inflammation of other organs around of bowels such as liver, spleen, kidney and bladder. The most cases of Gholonj are Inflammatory, Secretory, and Flatulency Gholonj. In these conditions occurs a dysfunction in intestinal tissue because of inflammatory process, mucoid phlegm or gas aggregation that leads to dysfunction of intestinal motility and passage of intestinal contents (6).

**Table 1:** Classification of intestinal obstruction (Gholonj) in view of Avicenna

**Type of Gholonj**	**Traditional term**	**Type of obstruction**	**Pathology**
Inflammatory Gholonj	Gholonj-e-varami	Non mechanical	Inflammation in the intestinal wall or surrounding organs
Secretory Gholonj	Gholonj-e-balghami	Non mechanical	Accumulation of mucoid Phlegm in lumen of intestine
Flatulency Gholonj	Gholonj-e-reehi	Non mechanical	High gas accumulation in the lumen of intestine
Fecal Gholonj	Gholonj-e-sofli	Mechanical	Accumulation of fecal masses
Parasitic Gholonj	Gholonj-e-doodi	Mechanical	Aggregation of worms in the lumen of intestine
Torsion Gholonj	Gholonj-e-eltevaee	Mechanical	Intestinal torsion or bowel herniation

Excluding type of mechanical obstruction, the strategy of treatment is based on medical because main pathology is no mechanical obstruction. For example, based on the definition of Gholonj-e-Reehi, Avicenna expresses that there is no any real obstruction agent; and accumulation of gas prevents from intestinal movement and defecation.

Medical treatment strategies of Golonj disease are various and includes of procedures, medical herbs, and diet recommendations.

Recent studies reveals pathophysiology of paralytic ileus is complex and multifactorial, consisting of neurologic and inflammatory agents. Activation of sympatic system or inflammatory response cells in muscle layers of bowel leads to generalized hypo motility of gastrointestinal tract (3). In explaining of physiopathology of non-mechanical obstruction Gholonj, Avicenna has written accumulation of material on the wall or lumen of intestinal and/or gas retention is the agent of non-passing the intestine contents (6). Avicenna despite unavailability to modern technology has represented some pathologies of intestinal obstruction that new study confirms them. Moreover, Avicenna has been mentioned some causes due to functional intestinal obstruction ignored in modern medicine.

Excluding some of Avicenna’s viewpoint and his etiology of intestinal obstruction based on humoral theories, most of his definition can be compared with medical current concepts. Increased insight into options mentioned in Qanun can be useful for new study, aiming to manage better of disturbances of gastrointestinal function. However, establishing of this concept requires further research and the generation of scientific evidence.
